# Structural bioinformatics for rational drug design

**DOI:** 10.1016/j.rpth.2025.102691

**Published:** 2025-01-23

**Authors:** Soroush Mozaffari, Agnethe Moen, Che Yee Ng, Gerry A.F. Nicolaes, Kanin Wichapong

**Affiliations:** 1 Department of Biochemistry, Cardiovascular Research Institute Maastricht (CARIM), Maastricht University, Maastricht, the Netherlands; 2 Hillmark B.V., Maastricht, the Netherlands

**Keywords:** artificial intelligence, computer-aided molecular design, drug discovery, machine learning, molecular docking, molecular dynamics simulation

## Abstract

A State of the Art lecture titled “structural bioinformatics technologies for rational drug design: from in silico to in vivo” was presented at the International Society on Thrombosis and Haemostasis (ISTH) Congress in 2024. Drug discovery remains a resource-intensive and complex endeavor, which usually takes over a decade and costs billions to bring a new therapeutic agent to market. However, the landscape of drug discovery has been transformed by the recent advancements in bioinformatics and cheminformatics. Key techniques, including structure- and ligand-based virtual screening, molecular dynamics simulations, and artificial intelligence–driven models are allowing researchers to explore vast chemical spaces, investigate molecular interactions, predict binding affinity, and optimize drug candidates with unprecedented accuracy and efficiency. These computational methods complement experimental techniques by accelerating the identification of viable drug candidates and refining lead compounds. Artificial intelligence models, alongside traditional physics-based simulations, now play an important role in predicting key properties such as binding affinity and toxicity, contributing to more informed decision-making, particularly early in the drug discovery process. Despite these advancements, challenges remain in terms of accuracy, interpretability, and the needed computational power. This review explores the state of the art in computational drug discovery, examining the latest methods and technologies, their transformative impact on the drug development pipeline, and the future directions needed to overcome remaining limitations. Finally, we summarize relevant data and highlight cases where various computational approaches were successfully applied to develop novel inhibitors, as presented during the ISTH 2024 Congress.

## 1 Introduction

**Figure 1:**
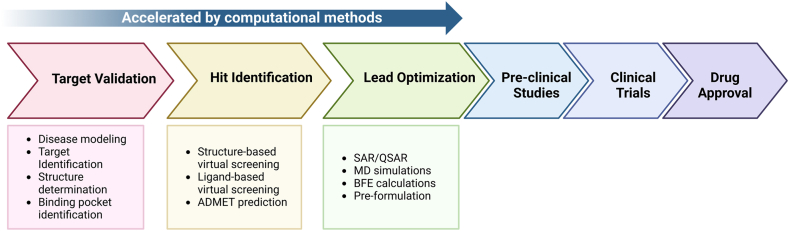
Drug discovery and development pipeline and application of computer/AI-based approaches in the early states to accelerate drug discovery campaigns. ADMET, absorption, distribution, metabolism, excretion, and toxicity; AI, artificial intelligence; BFE, binding free energy; MD, molecular dynamics; QSAR, quantitative structure–activity relationship; SAR, structure–activity relationship.

Drug discovery is an inherently challenging process, characterized by high costs, significant time investment, and a low success rate. It is estimated that bringing a new drug to the market can cost approximately $2 to 5 billion with timelines typically spanning 12 to over 15 years [1,2]. Furthermore, evolving regulatory demands and the complexities of clinical trials continue to drive costs higher. Much of these expenses and time could potentially be reduced by identifying high-quality hits and leads with superior selectivity and potency early in the drug discovery process (Figure 1), thereby increasing the likelihood of success.

The discovery of drugs and therapeutics has historically been a process mainly driven by trial-and-error experimentation and serendipitous findings. However, recent advancements in scientific knowledge have shifted this paradigm toward a rational drug design approach, which leverages an understanding of the biological mechanisms underlying diseases and the structure–activity relationships (SARs) of bioactive molecules (eg, inhibitors or activators). By utilizing this information, researchers can now systematically discover molecules with optimized properties for specific therapeutic effects, thereby improving the efficiency of drug development.

The rapid progress in computational technologies has been central to this transformation, enabling researchers to model and simulate complex biological and chemical systems at unprecedented levels. For instance, modern graphics processing units (GPUs) possess the capability to accelerate computational chemistry as, for example, in molecular dynamics (MD) simulations of biomolecules, providing valuable insights into the conformational changes and protein–protein/ligand interactions that underlie biological functions. This fundamental knowledge is essential for designing and optimizing bioactive compounds to achieve desired properties.

Moreover, machine learning (ML) and artificial intelligence (AI) techniques have emerged as powerful tools for capturing and processing complex patterns within masses of accumulated biological data over years, producing results with accuracy comparable to experimental methods [3,4]. This capability extends from predicting the three-dimensional (3D) structures of complex biomolecules to forecasting key properties of potential therapeutic agents, including binding affinity, toxicity, and other pharmacokinetic properties. Numerous studies have demonstrated remarkable success in accelerating various stages of drug discovery by application of AI/ML technology [[5], [6], [7], [8]]. Additionally, structural bioinformatics and cheminformatics apply the latest computational advances to facilitate rapid iterative optimization of chemical compounds prior to synthesis and experimental testing.

In this review, we will discuss the key aspects of computational drug discovery, beginning with the identification of initial hits and progressing through lead optimization. We will explore how the latest advancements in AI-driven structure prediction, virtual screening (VS), and MD simulations are transforming the landscapes of drug discovery. Additionally, we highlight the challenges that remain in integrating these techniques into a cohesive drug discovery pipeline, including issues of accuracy, model interpretability, and computational cost. Finally, we present cases, presented at the ISTH 2024 Congress, where various computational methods have been successfully employed to develop novel bioactive compounds (including small molecules, peptides, and therapeutic proteins) that target proteins involved in inflammation and atherosclerosis.

## 2 Identification of Bioactive Compounds: Experimental and Computational Methods

Discovering suitable compounds that exhibit favorable pharmacokinetic properties is a formidable task due to the vast chemical space available for exploration. It is estimated that the number of small molecules suitable for human administration is in the order of 10^60^ [9].

One of the traditional methods utilized to identify hit and lead compounds is high-throughput screening (HTS), which allows for rapid experimental evaluation of the biological activity of compound libraries against drug targets. Despite its experimental accuracy, the capacity of HTS is limited by the size of the compound libraries that can be screened, processing only up to 10,000 to 100,000 compounds at peak throughput [10]. This is insufficient to represent even a minuscule fraction of the available chemical space. According to Lyu et al. [11], screening ultralarge libraries, encompassing hundreds of millions of compounds, significantly enhances the chances of discovering novel chemotypes and more potent inhibitors. As the screened chemical space grows, the number of potential ligands increases exponentially, resulting in more compounds with high potency and selectivity. However, due to the limitations and costs associated with HTS, it is impractical to screen such extensive libraries in laboratory settings.

**Figure 2:**
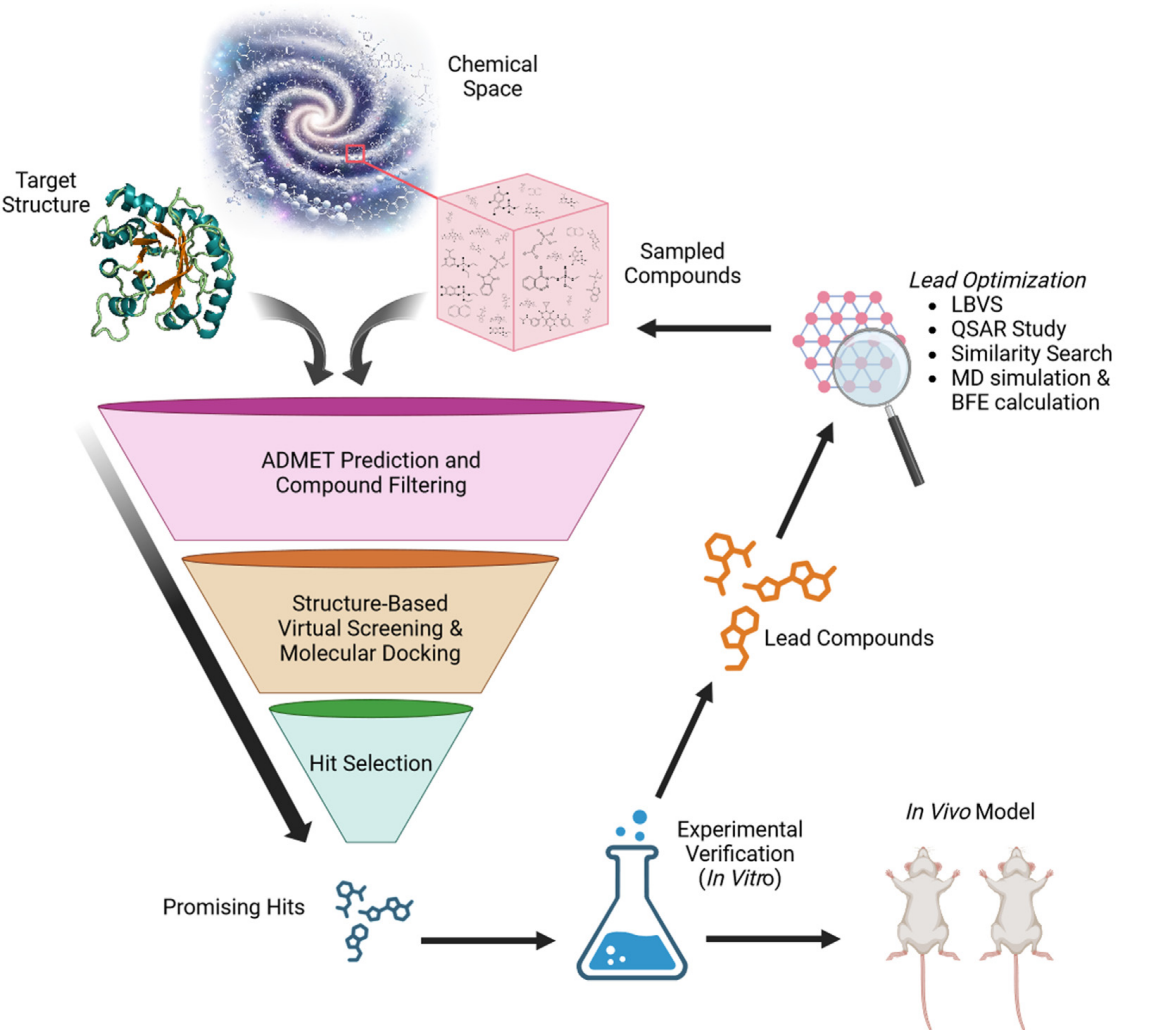
Schematic representation of an example of the application and integration of SBVS and LBVS in the drug discovery and development pipeline. ADMET, absorption, distribution, metabolism, excretion, and toxicity; BFE, binding free energy; MD, molecular dynamics; LBVS, ligand-based virtual screening; QSAR, quantitative structure–activity relationship; SBVS, structure-based virtual screening.

To address these limitations, the advent of computational techniques has paved the way for VS as a complementary approach. VS uses computational algorithms to evaluate and prioritize large compound libraries based on their predicted interactions with a target protein. There are 2 main types: structure-based virtual screening (SBVS), which includes the use of the 3D structure of target proteins, and ligand-based virtual screening (LBVS), which relies on known active (and inactive) compounds to identify new candidates. Leveraging increasingly accessible computational power, VS can explore vast chemical spaces beyond conventional HTS, efficiently narrowing down compounds likely to exhibit the desired biological activity. In the following sections, we will delve into the key steps of a general VS campaign (Figure 2) and provide an overview of the state of the art methods and technologies employed at each stage.

### 2.1 Determination of 3D structures of drug targets

To start with SBVS, the first essential step is to obtain the 3D structures of protein drug targets. Traditionally, X-ray crystallography and nuclear magnetic resonance (NMR) spectroscopy have been the main techniques for determining protein structures. X-ray crystallography involves the diffraction of X-rays through crystallized proteins, producing a diffraction pattern that can be interpreted to reveal the atomic structure. NMR spectroscopy, however, uses the magnetic properties of atomic nuclei to derive structural information, particularly for proteins in solution. In recent years, cryoelectron microscopy has emerged as a powerful alternative for protein structure determination. Cryoelectron microscopy involves flash-freezing protein samples and capturing images using electron microscopy. This method has gained prominence due to its ability to achieve resolutions comparable to X-ray crystallography and NMR, while often being easier to apply, particularly for large protein complexes that are challenging to crystallize [12], while requiring less protein sample than X-ray crystallography or NMR.

Despite these advances, as of this publication, only 17% of the total proteins in the human proteome have been characterized by experimental means. However, many of these experimental structures are often incomplete, lacking coverage of the entire protein sequence. To address this limitation, homology modeling has been employed, which involves predicting protein’s structures based on the known structures of closely related homologs [13]. While useful, this approach still has limitations, including the unavailability of accurate templates, challenges in loop modeling, and difficulties in quality assessment.

A significant breakthrough in protein structure prediction occurred in 2020 when AlphaFold2, developed by DeepMind, dramatically outperformed other methods in the 14th Critical Assessment of Protein Structure Prediction (CASP14) [3,4]. AlphaFold2 has revolutionized the structural biology field by utilizing AI technology to rapidly and accurately predict and generate 3D structures of biomolecules. The latest update, AlphaFold 3, further enhances this capability by integrating other chemical entities into its predictions, improving the accuracy and utility of the predicted structures [14]. Currently, more than 200 million protein structures have been created by AlphaFold, opening new avenues for researchers to expand drug targets to combat indomitable diseases by using these AI-based structures. Following AlphaFold’s success, other methods like RoseTTAFold have also been developed, further contributing to the availability of high-quality predicted structures [15]. These predicted structures are suitable for SBVS if they maintain a high confidence factor throughout the entire structure or, at least, at the binding pocket.

### 2.2 Identification of the binding pocket

While SBVS can be conducted without prior knowledge of the binding pocket, accurately identifying druggable and functionally relevant binding sites on protein structures is crucial for enhancing the efficiency of SBVS. These techniques are most effective when the binding sites are known beforehand. For targets where the binding pocket has not been experimentally identified and confirmed, such as through cocrystallization of the protein–ligand/substrate complexes, several computational methods have been developed to aid in identifying these sites.

Traditional methods like Fpocket [16], which relies on geometric analysis to identify potential binding pockets; FTSite [17] and Q-SiteFinder [18], which are energy-based approaches that predict binding sites by evaluating the interaction energies of probe molecules; and FINDSITE [19], which uses template-based predictions to infer binding pockets based on homologous structures. More recently, the advent of ML and deep learning techniques has led to the development of more sophisticated models. For instance, P2Rank [20] utilizes ensemble learning with a random forest algorithm to predict binding sites, while DeepPocket [21] and DeepSite [22] employ 3D convolutional neural networks to analyze protein structures. While the results are mixed, most of the AI-driven models have in general demonstrated improvements in accuracy and performance over conventional methods.

### 2.3 Absorption, distribution, metabolism, excretion, and toxicity prediction

Prediction of the absorption, distribution, metabolism, excretion, and toxicity (ADMET) properties of compounds is a critical step before conducting screening. ADMET predictions provide insight into the pharmacokinetic and drug-like properties of compounds, helping researchers identify candidates with the highest likelihood of success in biological environments.

Conventionally, *in silico* ADMET prediction methods typically employ quantitative structure–activity relationship and rule-based approaches. These methods predict ADMET properties by analyzing the relationships between the chemical structure and biological activity using molecular descriptors. Free web tools like SwissADME [23] are widely used. It combines empirical rules and ML algorithms to estimate important endpoints such as solubility, lipophilicity, and bioavailability. FAF-Drugs4 [24] is another free web tool that can be used to calculate the ADMET, as well as the physicochemical properties of compounds. Additionally, it includes a feature for filtering out pan assay interference compounds [25], enhancing the accuracy of identifying true hits. A comprehensive list and evaluation of free online ADMET prediction tools is available in the publication by Dulsat et al. [26]. More advanced AI/ML-based methods, such as graph convolutional networks and random forest, have shown great promise for predicting ADMET properties due to their capacity to handle complex, nonlinear relationships in large datasets [27]. Despite the superior performance of deep learning models in ADMET prediction, a significant challenge remains in the interpretability of these models. This lack of transparency makes it difficult to understand the specific structure–activity relationships that drive predictions, which can hinder their use in validating or rationally designing compounds to optimize desired properties.

### 2.4 SBVS and molecular docking

At the core of SBVS lies molecular docking, a computational process that predicts the binding pose and affinity of ligands with targets. Key challenges in this process are to accurately identify the most likely binding pose of the ligand and precisely predict binding affinity. Docking software addresses this challenge by balancing accuracy and speed. To achieve a balance, these tools often employ heuristic algorithms to reduce the search space, enabling faster calculations. Conventional docking programs such as AutoDock [28], AutoDock Vina [29], Glide [30], ICM Docking [31], FRED [32], Surflex-Dock [33], GOLD [34], and DOCK [35] exemplify this approach. Each software employs a distinct approach to managing the trade-off, with effectiveness varying based on the characteristics of the ligand and binding site.

An essential component of the docking software is the scoring function (SF), which estimates how strongly a ligand binds to the target. SFs are of high importance as they guide the docking process toward the most likely binding poses. Moreover, due to their fast calculations, SFs are frequently used to rank compounds after docking in large-scale screenings, helping prioritize and select candidates for further experimental validation. Numerous SFs have been developed by using different approaches, including physics-based methods (eg, AutoDock and DOCK score), knowledge-based techniques (eg, DrugScore [36]), empirical approaches (eg, ChemScore [34] and GlideScore [30]), and ML algorithms (eg, RF-Score [37]). The comparative assessment of the scoring function benchmark was established to evaluate 25 different SFs. The findings from comparative assessment of scoring functions-2016 offer useful insights that can help users make informed decisions when selecting scoring functions for docking and VS exercises [38].

Another important advancement in recent years is the adaptation of docking software for GPU computation, allowing for a significant acceleration of docking calculations. Examples of GPU-compatible docking programs include AutoDock-GPU [39], Vina-GPU [40], and Uni-Dock [41]. Among these approaches, Uni-Dock has demonstrated exceptional performance, achieving over 1000-fold speedup compared to single central processing unit core–based computations. This advanced technology enables researchers to explore vast chemical spaces and screen ultralarge databases (eg, the ZINC database [over 230 million compounds [42]] and the Enamine REAL database [approximately 6.75 billion compounds]). A study by Yu et al. [41] demonstrated that docking of 38.2 million ligands (Enamine Diverse REAL druglike set) can be completed within 12 hours by using Uni-Dock on a GPU cluster with 100 GPUs [41]. Furthermore, the adoption of AI/ML in docking software has not been limited to SFs and completely AI-based docking software (eg, EquiBind, DiffDock, and TANKbind) has also revolutionized by leveraging deep learning (DL) techniques to improve the efficiency of molecular docking [43]. A comparative study showed that traditional approaches, like Uni-Dock combined with pocket searchers such as P2Rank, consistently outperform DL models in docking tasks with the same pockets. However, DL models, particularly DiffDock, are approaching the accuracy of traditional methods and have potential to surpass them in the future as the models improve further in pocket searching and docking performance [43]. A significant concern with AI-based docking methods is the feasibility of the predicted binding poses. A study conducted by the creators of a software called PoseBusters [44] revealed that many predicted poses by AI models might not be physically realistic. Therefore, extra caution is required when interpreting the results from AI-based docking, as the rapid predictions might sometimes come at the cost of accuracy in determining viable binding poses. Furthermore, issues related to the reproducibility and transparency of AI models present additional challenges. One recent effort to address these concerns is the data optimization model evaluation Registry platform, which offers a standardized framework for reporting and curating ML studies in biological sciences. This initiative enhances transparency, reproducibility, and reliable validation of such studies [45].

### 2.5 LBVS

An alternative to SBVS is LBVS, which operates on the principle that molecules with similar structures tend to exhibit similar biological activities. LBVS can be of particular benefit when the structure of the target protein is not available, while there are known active (and/or inactive) compounds, or in ligand optimization contexts where the goal is to identify analogs of a lead compound with improved affinity or pharmacokinetic properties. In general, these methods utilize the chemical and structural features of known active (and/or inactive) compounds to establish quantitative structure–activity relationships and search libraries for new active compounds.

Similar to other areas, LBVS has also seen significant advancements with the integration of AI, where intricate patterns in the chemical and structural features of the ligand molecules can be learned by a given model to predict a single attribute or multiple ones on unseen molecules [46]. In a combined LBVS and SBVS, training a model on predicted binding affinities for large numbers of docked compounds facilitates the rapid identification of new binders from large compound libraries, often significantly reducing the time required compared to traditional molecular docking. This approach has achieved up to a 100-fold increase in the speed of SBVS by docking only a selected subset of the chemical library [5].

Meanwhile, these advancements are largely attributed to the development of efficient molecular representations that enable training ML and DL models [47]. A well-known example of these representations is the Simplified Molecular Input Line Entry System (SMILES), a notation that encodes the 2D structure of a chemical molecule as a text string [48]. However, the SMILES representation, despite being very efficient in terms of computation, comes with several limitations. One important drawback is that different molecules may share the same SMILES notation, leading to ambiguities [49]. Additionally, the SMILES format does not capture 3D spatial information, which can be crucial for understanding molecular interactions. To overcome these limitations, more accurate representations, such as graph-based representations (such as graph convolutional networks) [50] of molecules, have been developed and adopted. In these representations, atoms are treated as nodes and bonds as edges, capturing the molecular structure in a way that retains both connectivity and spatial relationships.

While LBVS is a powerful approach in drug discovery, it is not without limitations. One of the primary challenges is its dependence on the diversity and quality of the known active compounds used as templates. If these active compounds lack structural diversity, it may hinder the discovery of novel compounds that differ meaningfully from the existing ones, potentially limiting the efficient exploration of the chemical space.

### 2.6 MD simulations

One of the key challenges in computer-aided drug discovery is accounting for the inherent flexibility of biomolecules. Proteins, nucleic acids, and other biomolecules undergo continuous conformational changes that can influence their interaction with ligands. MD simulation is a computational technique used to simulate the motion of atoms and molecules over time. By calculating the forces between atoms and updating their positions step by step, MD allows to observe how a biomolecular system evolves, providing insights into the dynamic nature of biomolecules and their interactions with ligands.

While MD simulations can be applied in numerous contexts, 2 important applications are particularly valuable in VS campaigns. First, during the preliminary stages, before large-scale molecular docking, MD simulations can be used to explore the conformational flexibility of target biomolecules [51]. Second, MD simulations play a critical role in the later stages. Once hit molecules have been identified, MD simulations can be performed to gain detailed insights into protein–ligand interactions [52]. While molecular docking protocols typically rely on various assumptions, MD simulations are conducted under physiological conditions (eg, standard pH, temperature, and pressure) and incorporate essential factors such as solvation effects and flexibility, allowing them to better mimic biological systems.

**Figure 3:**
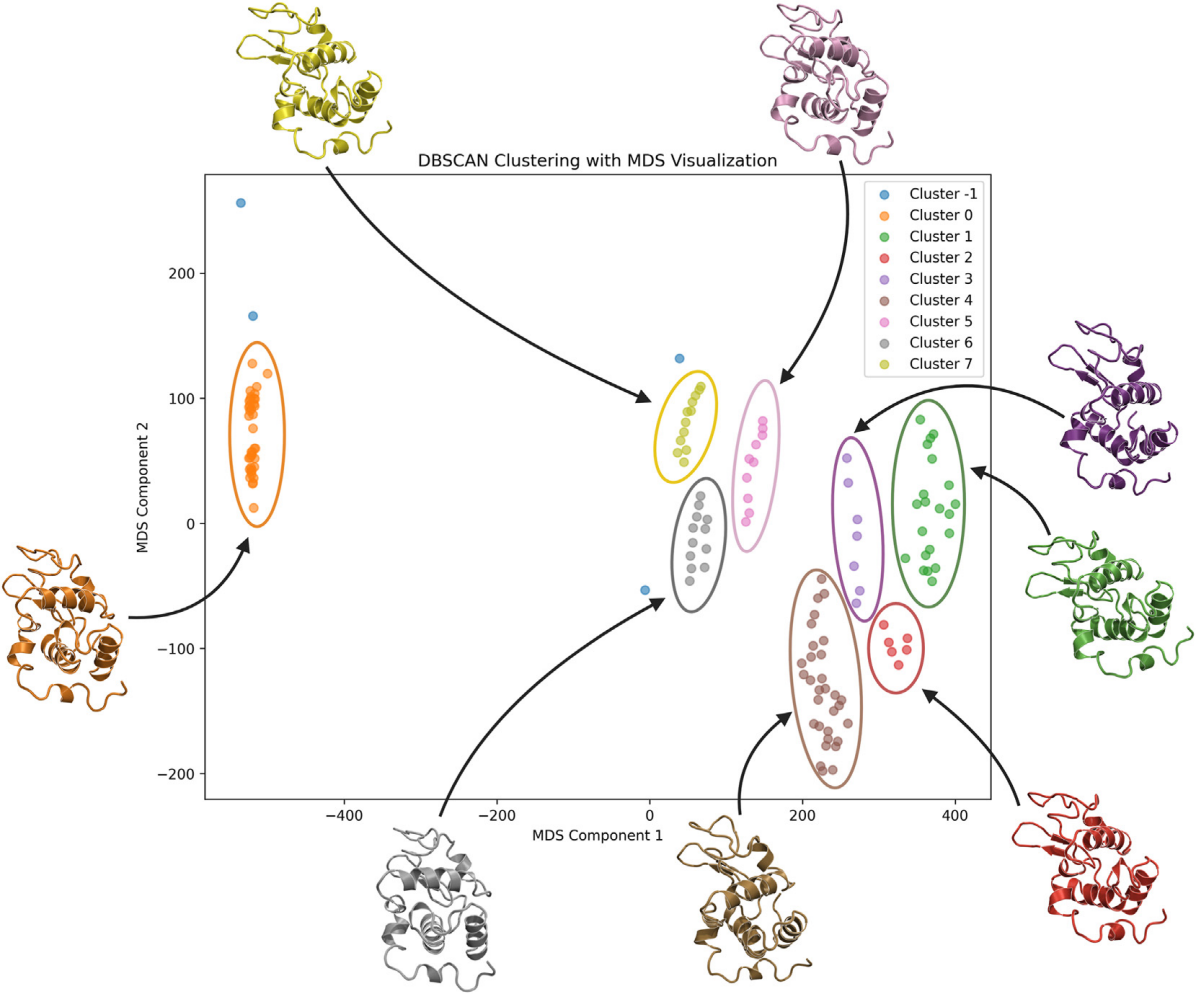
Protein conformations resulting from MD simulations are clustered using DBSCAN and visualized with MDS. Each point represents a conformation, and colors indicate distinct clusters. Cluster-1 marks outliers. DBSCAN, density-based spatial clustering of applications with noise; MD, molecular dynamics; MDS, multidimensional scaling.

To explore conformational spaces of target biomolecules, MD simulations can be employed to capture their flexibility, including the movement of loops, side chains, and secondary structures. In addition to the traditional MD simulations, enhanced sampling techniques such as metadynamics, replica exchange molecular dynamics [53], Gaussian-accelerated molecular dynamics [54], and hydrogen mass repartitioning MD [55] have been developed. These methods allow the system to explore a broader conformational space within a feasible simulation time, increasing the chances of capturing functionally relevant conformations. MD trajectories represent a time-resolved series of molecular snapshots, each representing a specific molecular structure for analysis and comparison. Common structural comparison methods include root mean square deviation, which quantifies the overall deviation from a reference structure, and root mean square fluctuation, which measures per-atom/residue variations to identify flexible regions. All-vs-all comparisons of snapshots offer a comprehensive view of conformational space but often result in high-dimensional data. Dimensionality reduction techniques, such as principal component analysis (PCA) and multidimensional scaling [56], help visualize this space in lower dimensions, revealing dominant structural trends. Additionally, combining PCA with free energy landscape calculations and clustering analysis can be employed to identify stable conformational states, offering insights into protein dynamics and flexibility [57]. Moreover, clustering algorithms like *K*-means [58] or density-based spatial clustering of applications with noise [59] can be used to further group snapshots into different clusters based on their relative structural similarity (Figure 3). These representative structures, or snapshots, offer a more inclusive view of the conformational space and potential binding sites for subsequent molecular docking experiments. This approach, often referred to as ensemble docking, allows to account for the flexibility of the target, leading to more accurate and realistic predictions of ligand binding [60]. Docking ligands into multiple conformations obtained from MD simulations enables researchers to identify which ligands are most likely to bind across different structural states, providing a more holistic view of ligand–target interactions.

In the context of hit-to-lead translation, once ligands are identified as potential binders through VS or other techniques, MD simulations can refine our understanding of the protein–ligand interactions. As mentioned above, unlike static molecular docking, which assumes a rigid binding site, MD allows for dynamic adaptation of both the ligand and the target, and the interplay between them, capturing conformational changes that enhance the accuracy of predicted binding poses. For example, side chains of residues in the binding pocket may change their conformations to accommodate ligand, or the ligand itself may adjust its conformation to optimize interactions. Moreover, analysis of MD trajectories can yield valuable insights into key interacting residues, significant interactions (eg, hydrogen bonds, salt bridges, and hydrophobic contacts), and the stability of the complex. This critical information can be utilized to guide the optimization process for designing ligands with desired properties.

Furthermore, MD simulations facilitate more accurate binding affinity predictions through free energy calculations [61]. Methods such as molecular mechanics Poisson–Boltzmann (generalized Born) surface area can be applied to MD trajectories to estimate binding free energy (BFE) [62]. Another rapid end-point method that can be used to estimate BFE of protein–ligand complexes is linear interaction energy (LIE), which necessitates the empirical fitting of scaling parameters [63]. Recently, several ML algorithms have been integrated with MD simulations to develop novel BFE approaches, eg, 3D-RISM-AI [64] and ANI_LIE (combining LIE with ANI-2x neural network) [65], offering enhanced accuracy and speed. These calculations provide more precise insights into binding affinity than docking scores alone, aiding in the prioritization of potential lead compounds. As previously demonstrated, the use of BFE methods can significantly reduce the number of candidate compounds for further synthesis and experimental evaluation, thereby accelerating the hit/lead identification process [66,67].

By integrating these key aspects, researchers can use a generic MD pipeline to develop novel bioactive compounds targeting drug targets. Briefly, 3D structures, derived from experimental or computational methods, are required. During the preparation phase, key parameters such as suitable force fields for proteins/peptides, lipids, RNA/DNA, and organic compounds are assigned using MD software. The systems are then solvated with water molecules and counter ions and then subjected to MD simulations. After the simulation, MD snapshots are analyzed using, for example, root mean square deviation, root mean square fluctuation, PCA, and clustering analysis approach to assess flexibility and identify stable conformational states. Residue interaction networks involved in ligand binding or conformational modulation can be identified through per-residue energy decomposition (DC) and hydrogen-bond occupation analysis. Also, BFE calculations can be employed to predict and prioritize the binding affinity of designed compounds. By using this pipeline, we have successfully developed small molecules [68], peptides [66,69], and therapeutic proteins [70,71] targeting various proteins.

With the advent of high performance of software and hardware, particularly, the use of GPUs has dramatically accelerated MD simulations, enabling the exploration of more complex biological systems at longer timescales [72]. Popular software packages such as GROMACS [73], AMBER [72], Yasara [74], and NAMD [75] are optimized for GPU-based computing, significantly enhancing their performance. The development of faster algorithms, enhanced sampling methods, and more accessible computational resources is necessary to make MD simulations more practical for routine use in large-scale drug discovery campaigns.

### 2.7 Application of computational approaches to develop bioactive compounds with anti-inflammatory and antiatherosclerotic activities

The concept of computer-aided drug discovery was introduced in the 1970s [2], and since then, numerous drug candidates have been developed using computational and AI techniques. Several of these candidates have advanced to clinical trials or have been developed into drugs for clinical use [76,77]. In this section, we highlight some selected cases and discuss how various computational methods were utilized to develop bioactive compounds targeting different drug targets.

#### 2.7.1 Small-molecule inhibitors

CD40 is a transmembrane protein receptor that, upon interacting with its ligand (CD40L), triggers inflammatory responses. These responses can contribute to the development of chronic inflammatory diseases, such as atherosclerosis. Inhibition of CD40L may not represent a viable therapeutic target since prolonged blockage could weaken systemic immune responses. A pioneering study by Lutgens et al. [78] revealed a novel and promising approach to regulating this immunological pathway by blocking the interactions between CD40 and tumor necrosis factor receptor–associated factor 6 (TRAF6).

**Figure 4:**
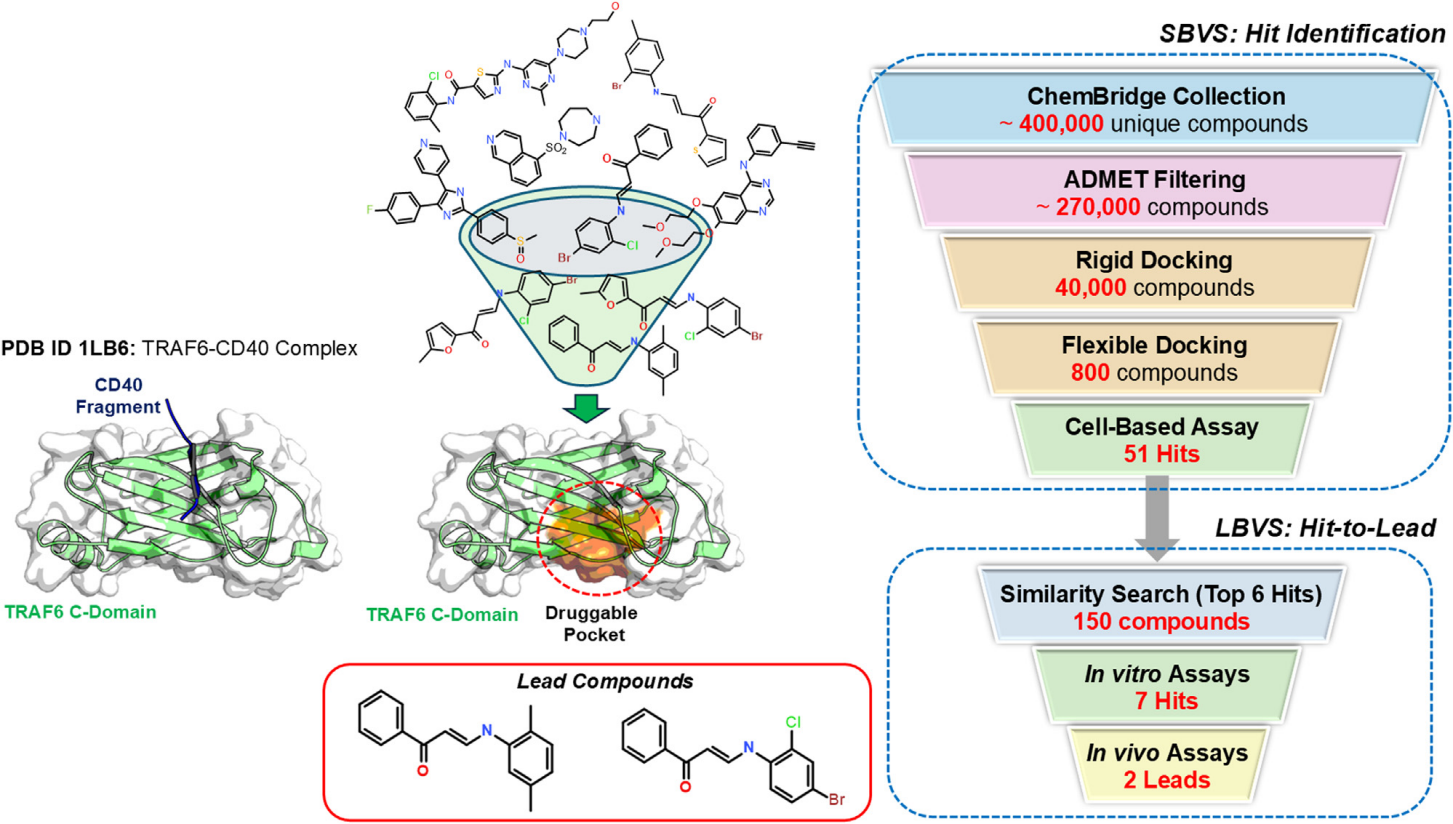
Schematic representation of SBVS and LBVS to identify and develop novel TRAF6 small-molecule inhibitors. ADMET, absorption, distribution, metabolism, excretion, and toxicity; LBVS, ligand-based virtual screening; PDB, Protein Data Bank; SBVS, structure-based virtual screening.

To develop small-molecule inhibitors to disrupt the CD40–TRAF6 interactions, SBVS and LBVS were employed [79,80] (Figure 4). The 3D structure of CD40–TRAF6 complex (Protein Data Bank ID: 1LB6) and the Express Pick ChemBridge database (version November 2009), which consisted of approximately 400,000 compounds, were used. The database was first filtered based on the ADMET properties of the compounds using Lipinski’s rule of 5, using the FAF-Drugs2 online tool. Compounds with one Lipinski violation or those containing reactive groups were excluded, leaving approximately 270,000 compounds for further docking. Next, multiple steps of molecular docking were performed, including rigid (FRED [32]) and flexible docking (Surflex-Dock [33]). The docking results were ranked based on their scores, and the top 800 compounds were purchased and subsequently tested in a cell-based luciferase assay to evaluate their ability to influence TRAF6-dependent NF-κB formation. Among these 800 compounds, 51 compounds were identified as hits, exhibiting over 50% inhibition at a single concentration of 10 μM. Additionally, 6 of these 51 compounds showed greater than 90% inhibitory activity. Next, LBVS was conducted using the top 6 hit compounds as templates to search for similar compounds in the ChemBridge database (Hit2Lead) with a Tanimoto coefficient cut-off of 0.80. Using LBVS, 150 similar compounds were retrieved and subjected to experimental validation, including *in vitro*, cell-based assays and direct binding measurements. The top 7 hit compounds, exhibiting over 95% inhibition at a single concentration of 10 μM, were identified. Finally, 2 compounds with *in vitro* IC_50_ values between 0.1 and 16 μM were selected for *in vivo* studies, and they exhibited therapeutic benefits in various animal models, including sepsis, obesity, and neuroinflammation [[79], [80], [81], [82], [83]].

Furthermore, similar approaches were also successfully applied to identify and develop novel inhibitors for coagulation factors V [84] and VIII [85], activated protein C (APC) [86], and neutral sphingomyelinase 2 (nSMase2 [87]), just to name a few, with the former being the first small-molecule inhibitor of a protein–membrane interaction discovered through computational techniques.

#### 2.7.2 Peptide inhibitors

Peptides are biopolymers, consisting of 2 to 50 amino acids linked by amide bonds. In recent years, peptide-based drugs have experienced a resurgence in popularity. Since the development of insulin, the first therapeutic peptide, in 1921, significant advancements have led to the approval for use as therapeutics of over 80 peptide drugs globally [88].

**Figure 5:**
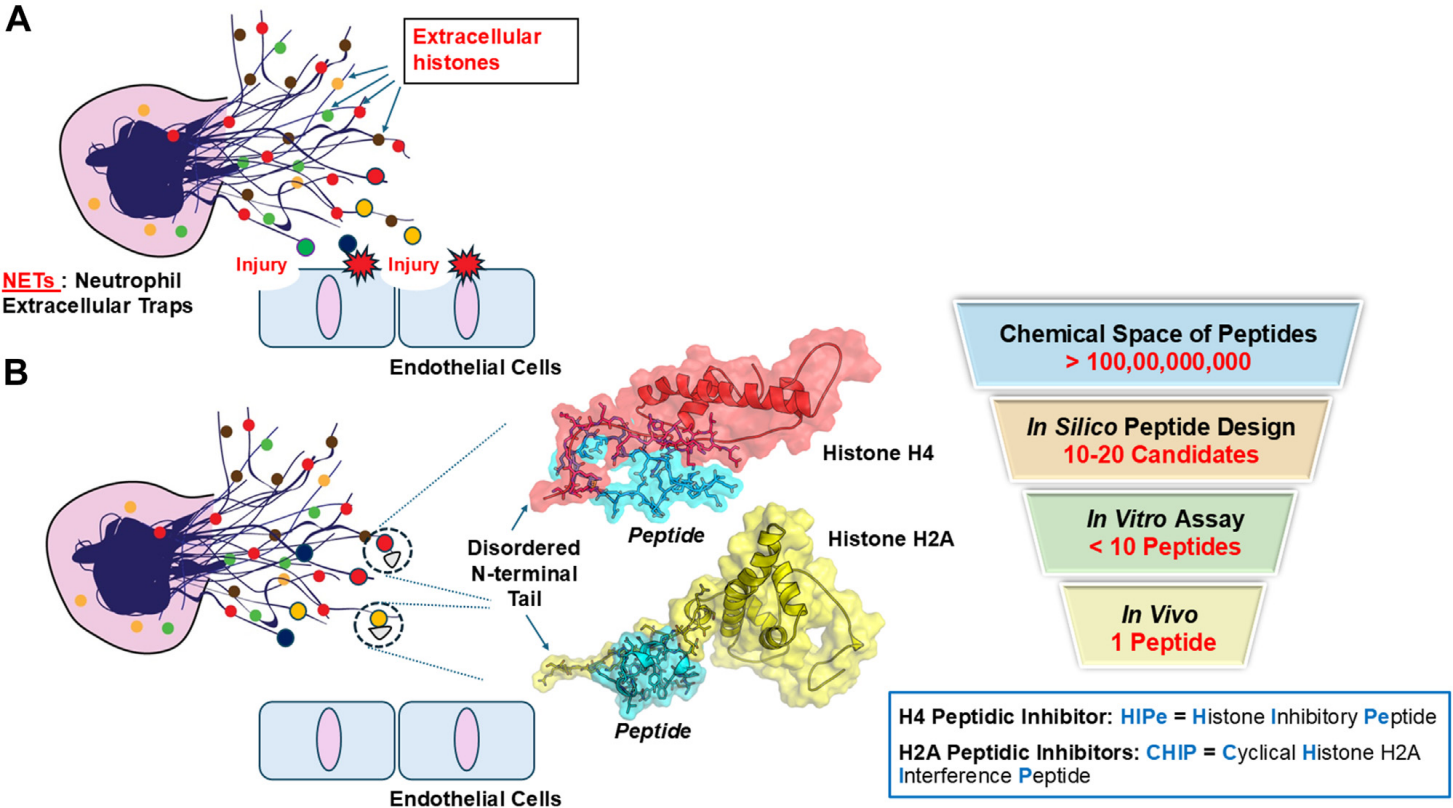
(A) Extracellular histones released from NETs exhibit cytotoxicity and induce cell injury. (B) Neutralization of histone cytotoxicity by peptides, developed through structure-based approaches, can prevent cell injury. A hierarchical strategy in the development of peptidic inhibitors targeting extracellular histones (H2A and H4) is demonstrated.

In 2009, Xu et al. [89] discovered the cytotoxic properties of extracellular histones, which can lead to organ injury and eventually death. Since then, the role of histones in the pathogenesis of inflammatory diseases, cardiovascular diseases, sepsis, and COVID-19, has been extensively investigated [[90], [91], [92], [93], [94]]. Unlike TRAF6, which contains a well-defined druggable pocket, the N-terminal domains of histones, the primary domain responsible for activating cell death, are considered disordered regions. Therefore, peptides or therapeutic proteins capable of binding to and capturing the unstructured tails of histones represent a promising approach for neutralizing these histones. To develop peptidic inhibitors targeting histone H2A/H4, we employed a structure-based approach [69] (Figure 5). Briefly, complexes between histone H2A/H4 and their binding partners, either derived from experimental structures in the Protein Data Bank or generated through protein–protein docking, were utilized as starting structures and subjected to MD simulations. Next, key interacting residues were identified and extracted from the binding partners to generate a template peptide. Rational peptide design was then conducted by performing *in silico* mutations of specific residues to enhance interactions between the peptides and histone H2A/H4. A series of peptides (10-20 candidates) were designed *in silico*, and their binding affinities with histone H2A/H4 were predicted using BFE calculations. Subsequently, fewer than 10 peptides with low BFE values were selected for synthesis and *in vitro* testing. The most potent compound (1 peptide for target) was chosen for *in vivo* testing, which demonstrated potential therapeutic benefits in atherosclerotic mouse models [95,96].

The structure-based approach described here has also been applied to develop peptides targeting various proteins involved in coagulation and inflammation, including CCL5-HNP1 [66,97], GPIbα-VWF A1 [98], and CIB1-integrin αIIbβ3 [99].

#### 2.7.3 Therapeutic proteins

A recombinant human activated protein C (APC), known as Xigris, was approved for the treatment of severe sepsis and used from 2001 to 2011. It was withdrawn from the market after studies showed no significant reduction in mortality in severe sepsis patients and that it is linked to a higher risk of bleeding [100]. A study by Xu et al. [89] demonstrated that APC exhibited cytoprotective properties by binding to extracellular histones and cleaving them, thereby reducing their cytotoxicity. To develop new APC variants with reduced anticoagulant activity but improved binding and proteolytic properties for histones, Huckriede et al. [71] utilized a structure-based method, combining protein–protein docking, MD simulations, and BFE calculations, to investigate interactions between wild-type (WT) APC and human histone H3. Given that APC contains 461 amino acid residues, there are at least 20^461^ possibilities for randomly mutating and producing APC variants. However, by using computer-based methods, only 3 new APC variants were designed *in silico*, experimentally purified, and tested for activity. The new variants exhibited decreased anticoagulant activity while still retaining the ability to proteolyze histone H3 with improved binding properties.

## 3 Conclusion

The landscape of drug discovery and development has been fundamentally transformed by advancements in computational technologies, offering new opportunities to mitigate the inherent challenges of high costs, prolonged timelines, and low success rates. Key innovations, such as VS, MD simulations, and AI-driven approaches, have significantly enhanced the capacity to explore vast chemical spaces, predict molecular interactions, and optimize drug candidates with improved efficiency and accuracy. SBVS and LBVS now leverage the increasing availability of high-quality protein structures, advanced docking algorithms, and deep learning models to identify promising therapeutic candidates more rapidly than traditional methods. MD simulations further refine these predictions by accounting for the dynamic nature of biomolecular systems, allowing for more realistic and accurate assessments of ligand–target interactions.

Furthermore, we demonstrate the use of computational methods to aid in the development of novel bioactive compounds. These methods are quite generic; therefore, they can be widely employed for various drug targets. Importantly, we have clearly demonstrated that by utilizing computer-based methods, we can significantly reduce the number of compounds tested in the lab, which can also accelerate the drug development process while leaving a lower environmental footprint.

However, despite these advances, challenges remain. The accuracy of predictions is still constrained by the quality of computational models, limitations in sampling timescales, and the availability of high-quality experimental data. AI-based models, though powerful, often lack transparency, posing interpretability issues that hinder trust in their predictions. Additionally, scaling these technologies to address the entire chemical space and biological complexity requires further improvements in computational efficiency and hardware capabilities.
